# For a Colorful Life: Recent Advances in Anthocyanin Biosynthesis during Leaf Senescence

**DOI:** 10.3390/biology13050329

**Published:** 2024-05-09

**Authors:** Ziqi Pei, Yifei Huang, Junbei Ni, Yong Liu, Qinsong Yang

**Affiliations:** 1 State Key Laboratory of Efficient Production of Forest Resources, Beijing Forestry University, Beijing 100083, China; pzq1667928733@163.com (Z.P.); 18950679107@163.com (Y.H.); lyong@bjfu.edu.cn (Y.L.); 2 Research Center of Deciduous Oaks, Beijing Forestry University, Beijing 100083, China; 3 Key Laboratory for Silviculture and Conservation, Ministry of Education, Beijing Forestry University, Beijing 100083, China; 4 College of Agriculture and Biotechnology, Zhejiang University, Hangzhou 310058, China; nijunbei@zju.edu.cn

**Keywords:** leaf senescence, anthocyanin, MYB, environmental factors, signaling pathways

## Abstract

**Simple Summary:**

Leaves often turn red during senescence, providing us with a colorful life. Anthocyanin accumulation is the main cause of the coloration and its biosynthesis during leaf senescence is an important biological process, which might contain different mechanisms from other tissues. It is essential to understand the molecular mechanism of anthocyanin accumulation during leaf senescence, which would provide new insight into leaf coloration and molecular breeding for more colorful plants in spring or autumn. In this review, we focused on leaf coloration during senescence. We emphatically discussed several networks linked to genetic, hormonal, environmental, and nutritional factors in regulating anthocyanin accumulation during leaf senescence. This paper aims to provide a regulatory model for leaf coloration and to put forward some prospects for future studies.

**Abstract:**

Leaf senescence is the last stage of leaf development, and it is accompanied by a leaf color change. In some species, anthocyanins are accumulated during leaf senescence, which are vital indicators for both ornamental and commercial value. Therefore, it is essential to understand the molecular mechanism of anthocyanin accumulation during leaf senescence, which would provide new insight into autumn coloration and molecular breeding for more colorful plants. Anthocyanin accumulation is a surprisingly complex process, and significant advances have been made in the past decades. In this review, we focused on leaf coloration during senescence. We emphatically discussed several networks linked to genetic, hormonal, environmental, and nutritional factors in regulating anthocyanin accumulation during leaf senescence. This paper aims to provide a regulatory model for leaf coloration and to put forward some prospects for future development.

## 1. Introduction

Anthocyanins are water-soluble pigments produced in the cytoplasm of plants and are widely distributed in plant organs such as fruits and leaves. They impart vibrant colors to various organs, especially flowers and fruits, aiding in the attraction of seed dispersers. Anthocyanins serve as secondary metabolites, protecting plants from both biotic and abiotic stresses [1]. Additionally, they possess the ability to scavenge free radicals and exhibit antioxidant properties. This capability not only helps living organisms defend against oxidative damage, but also provides various beneficial health effects for humans [2,3]. Anthocyanins and their derivatives are recognized for their roles in protecting eyesight and slowing brain aging [2], leading to their widespread inclusion in daily diets. Apart from their health benefits, there is a growing interest in utilizing anthocyanins to augment the aesthetic qualities of plants, thereby elevating their ornamental value. Consequently, breeding programs now prioritize the creation of anthocyanin-enriched plants.

In this review, the genetic regulation of anthocyanins’ biosynthesis is elaborated, and the phytohormones and environmental regulation of biosynthesis is discussed subsequently. By summarizing existing knowledge, we concluded the regulatory network and factors affecting anthocyanin accumulation during leaf senescence. The review can provide valuable insights for developing strategies for obtaining colored-leaf trees through breeding and controlled environmental conditions.

## 2. Anthocyanin and Its Biosynthesis

A comprehensive understanding of anthocyanin is helpful to target regulation of anthocyanin accumulation in plants. Over the past few decades, a great deal of work has been performed to reveal the characteristics and synthetic mechanisms of anthocyanin. Anthocyanins belong to the flavonoids compound with a typical structure that has C6-C3-C6 as the basic skeleton. All the natural anthocyanins exist in the form of glycosides. There are six types of anthocyanins commonly found in plants: delphinidin 3-*O*-glucosides, cyanidin 3-*O*-glucosides, pelargonidin 3-*O*-glucosides, peonidin 3-*O*-glucosides, petunidin 3-*O*-glucosides, and malvidin 3-*O*-glucosides [4]. The color of anthocyanins changes depending on the pH, co-existing colorless compounds, and metal ions [5]. In acidic conditions, anthocyanins appear as red but turn blue when the pH increases. Metal ions, such as Al^3+^ and Fe^3+^, play a critical role in the generation of blue flowers in *Tulipa gesneriana* and *Hydrangea macrophylla* [6,7].

### 2.1. Biological Functions of Anthocyanins

At present, a large number of in vivo and in vitro experiments have proved that anthocyanin has many functions, such as antibacterial, anti-inflammatory, anticancer, antioxidant, free radical scavenging, and the prevention of cardiovascular diseases. Previous studies on the antioxidant properties of six deoxyanthocyanins showed that cyanidin-3-glucoside showed high anti-free radical and reductive activity in DPPH and FRAP experiments [8]. In addition, anthocyanin is also helpful for cancer prevention and treatment, such as colon cancer, liver and bladder cancer, breast cancer, and so on [9,10,11]. The anthocyanin metabolites gallic acid and 3-O-methylgallic acid can inhibit cell proliferation and induce cell apoptosis at the same time to achieve anticancer effects [12]. Moreover, anthocyanins are able to fight inflammation [13] and contribute to eye health [14]. Due to the powerful biological functions of anthocyanins, fruits and vegetables rich in anthocyanins are widely considered to be beneficial to the human body. Anthocyanins are widely used for their health care functions. Not only that, anthocyanin, as one of the important pigments of ornamental plants, creates a colorful life for people [6,7].

### 2.2. Biological Metabolic Pathways of Anthocyanins

Anthocyanin synthesis is a complex process, which is synthesized by the phenylpropyl pathway. Many studies have collectively demonstrated that the process is conservatively co-catalyzed by phenylalanine ammonia lyase [15,16], cinnamic acid 4-hydroxylase (C4H), 4-coumarate-CoA ligase (4CL), chalcone synthase (CHS), chalcone isomerase (CHI), flavanone 3-hydroxylase (F3H), dihydroflavonol 4-reductase (DFR), anthocyanidin synthase (ANS), and UDPG-flavonoid glucosyltransferase (UFGT) [2,17]. Various mutations in these anthocyanin biosynthesis genes lead to abnormal anthocyanin pigmentation in plants. For example, splicing changes in the promoter region of DFR gene in eggplant (*Solanum melongena*) lead to abnormal coding of dihydroflavonol 4-reductase and affect anthocyanin accumulation [18]. After synthesis and modification in the cytoplasm and endoplasmic reticulum membranes, anthocyanins are transported to the vacuole by the action of glutathione transferase (GST). Results showed that two loss-of-function alleles in the GST gene cause early termination of the translation and anthocyanin deficiency in the flower and fruit skin of peach [19].

### 2.3. MYB-Centered Molecular Network

The biosynthesis of plant anthocyanins is not only controlled by structural genes, but also influenced by regulatory genes and other factors. It is generally believed that the MBW complex formed by R2R3-MYB, bHLH, and WD40, is an important regulatory complex for anthocyanin biosynthesis, with MYB TFs playing a central role [20,21]. In kiwifruit (*Actinidia chinensis*), *AcMYB10* and *AcMYB110* act as core transcriptional activators, promoting anthocyanin accumulation in kiwifruit pulp [22]. Multiple MYB members, such as *MdMYB1* in apple (*Malus domestica*), co-regulate anthocyanin pigmentation [23,24,25]. And yet the insertion of a Long Terminal Repeat Transposable Element (LTR-TE) in the exon of *FvMYB10* lead to different anthocyanin accumulation in the skin and flesh of a diploid strawberry [26]. Moreover, the insertion of a 288-bp in the promoter of ReS (*GhMYB113*) enhances anthocyanin accumulation in cotton (*Gossypium hirsutum*) resulting in red foliated cotton [27]. A single nucleotide substitution of 10 bp upstream of the start codon in the R2R3-MYB gene *PETAL LOBEANTHOCYANIN* (*PELAN*) in *Mimulus* leads to the loss of protein function and inhibition of anthocyanin accumulation [28]. In recent years, with in-depth research, it has been found that MYB transcription factors also play a decisive role in leaf coloring. *ApMYB1* acts as a positive regulator during leaf coloration in ornamental plant *Acer palmatum* [29]. With the accumulation of anthocyanin, the expression of *PcMYB113* increases in *Pistacia chinensis* during leaf senescence [30]. Further results of functional verification confirmed that overexpression of *PcMYB113* could promote anthocyanin accumulation in *Arabidopsis thaliana*. Under lower temperatures and changing light conditions in autumn, the leaf coloring of two oak species was attributed to the marked upregulation of *QdMYB* in *Quercus dentata*, and the significantly higher expression of *QaMYB1* and *QaMYB3* in *Q. aliena* during senescence [31,32]. Formosan sweet gum (*Liquidambar formosana*) is a deciduous tree with dark red autumn leaves and purple young leaves. The different colors are attributed to the different regulations of MYB TFs, LfMYB5 increases the expression of *LfF3′5′H*, LfMYB123 induces the expression of *LfF3′H1* and *LfDFR1* in the spring, while LfMYB113 up-regulates the expression of *LfF3′H1*, *LfDFR1*, and *LfDFR2* in late autumn during leaf senescence [33]. Therefore, we speculate that some MYBs in plants can conservatively promote anthocyanin accumulation.

Apart from anthocyanin-activating MYBs, some MYBs are also involved in inhibiting anthocyanin accumulation in various ways. First of all, most MYB inhibitors have repression motifs in the C-terminal such as C1 (LIsrGIDPxT/SHRxI/L), EAR (LxLxL or DLNxxP), or TLLLFR [34]. LvMYB1, as a negative MYB factor, inhibits anthocyanin synthesis in lily (*Lilium* spp.) through its EAR motif [35]. In addition, the MYB inhibitor can compete with the MYB activator for binding to basic Helix Loop Helixes (bHLHs), thereby reducing the accumulation of anthocyanin. In peaches (*Prunus persica*), PpMYB18 protein competes with PpMYB10 to interact with PpbHLH3 and PpbHLH33, resulting in reduced anthocyanin accumulation in the peel [36]. In addition, IbMYB44 in purple-fleshed sweet potato could inhibit the MYB340-bHLH2-NAC56 complex, which negatively affects anthocyanin synthesis [37]. Overall, MYB transcription factors are essential to the regulatory network that regulates the production of anthocyanins across multiple organs.

**Figure 1:**
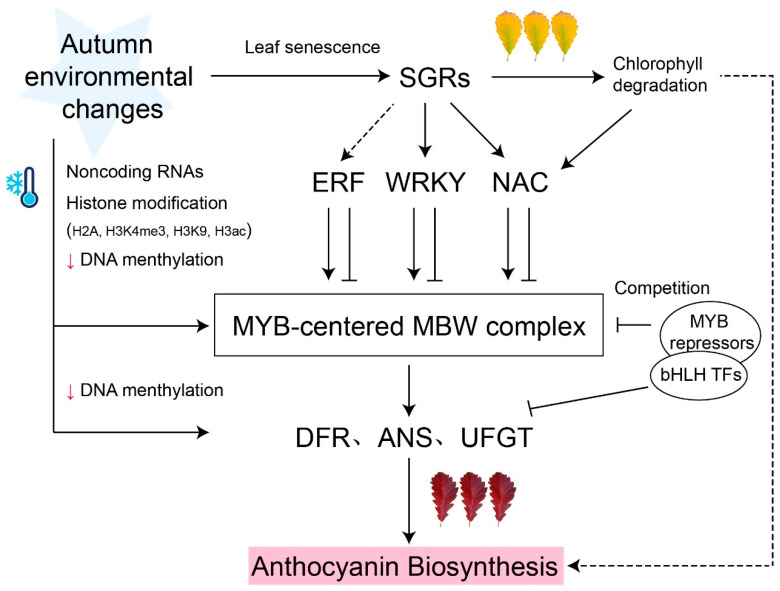
Model of MYB-centered molecular network of anthocyanin biosynthesis. Anthocyanin biosynthesis transcription is regulated by the MYB-centered MBW complex. Several MYB repressors competitively bind to bHLH TFs and negatively regulated anthocyanin biosynthesis. Other transcription factors (e.g., ERF, WRKY, and NAC) induced by STAY-GREEN (SGR) act upstream of MYB-centered MBW complex, activating or inhibiting anthocyanin accumulation. Epigenetic regulations, such as DNA methylation, histone H2A, H3K4me3, H3K9, and H3ac modification, participate in the regulation of MYB-centered anthocyanin biosynthesis. Black arrows represent activation; ‘T’ arrows represent repression; small red arrows represent a level decrease. Dashed lines denote indirect regulation or uncertain pathways.

Other transcription factors including HY5, BBX, NAC, and WRKY have been reported to play key roles in anthocyanin accumulation by regulating the expression of MYBs (Figure 1). *FvRIF*, a NAC transcription factor, activates the transcription of *FvMYB10* in the fruit of strawberry, establishing a clear connection between fruit development and anthocyanin accumulation [38]. Several BBX proteins in pears have been identified as promoters of anthocyanin accumulation. PpBBX18 and PpBBX16 form heterodimers with PpHY5 through two B-box domains, thereby activating the *PpMYB10* expression and promoting anthocyanin accumulation in the peel of red pears [39,40]. Additionally, A 14-bp deletion in *PyBBX24* causes premature translation termination, preventing *PyMYB10*-induced anthocyanin accumulation in pears [41]. MdWRKY75 in apple peel binds to the promoter of *MdMYB1* to stimulate the accumulation of anthocyanins [42]. In the bright red autumn leaves of *Q. dentata*, QdNAC may regulate anthocyanin accumulation and chlorophyll degradation during leaf senescence through direct interaction with QdMYB [32].

In recent years, increasing evidence has suggested that noncoding RNAs also play important roles in anthocyanin biosynthesis (Figure 1). In *M. spectabilis* leaves, miR858 negatively regulates *MsMYB62-like*, an anthocyanin biosynthesis inhibitor, and promotes anthocyanin accumulation under low-nitrogen conditions [43]. Similarly, miR156 targets to the *SQUAMOSA PROMOTER BINDING PROTEINLIKE* (*SPL*), destabilizing the MBW complex and inhibiting anthocyanin biosynthesis at the junction between the rosette and the stem in *Arabidopsis* [44].

### 2.4. Epigenetic Regulations

Epigenetic modifications, including DNA methylation and histone modification, are increasingly being shown to be involved in the manufacture of anthocyanins. The regulatory role of DNA methylation has been shown in many plants to be involved in the biosynthesis of anthocyanin. For example, different methylation intensities on the promoter of the *ANS* gene result in red and white colors in different lotus (*Nelumbo nucifera*) cultivars [45]. In many fruits, the methylation level of the *MYB10* promoter region is negatively correlated with peel color and anthocyanin accumulation [46,47]. Additionally, environmental factors have also been shown to affect DNA methylation levels. Bai and Tuan [48] showed that shading treatment decreased the methylation level of *MdMYB1-2/-3* promoters, initiated anthocyanin biosynthesis, and significantly increased the pigment content of non-red apple varieties. Low temperature induces the accumulation of anthocyanin and promotes leaf coloring by decreasing the methylation level of promoters in structural genes, which has been confirmed in all three varieties of *M. domestica* [49]. In conclusion, the DNA methylation level of the promoter in anthocyanin biosynthesis gene is closely related to the accumulation of anthocyanin.

Histone modifications are also evidently important in regulating anthocyanin biosynthesis. In *Arabidopsis*, the deposition of H2A.Z, a histone H2 variant, downregulates the expression of several genes related anthocyanin biosynthesis by inhibiting trimethylation of lysine 4 on histone H3 (H3K4me3), thereby preventing anthocyanin accumulation between the hypocotyl and cotyledons [50]. In poplar, a conserved histone H3K9 demethylase, JMJ25, directly binds to the negative transcription factor *PtrMYB182* gene loci and upregulates its expression, thereby inhibiting anthocyanin biosynthesis in leaves [51]. Histone acetylation can also affect the production of anthocyanin. In pear fruit, the PpERF9-PpTPL1 complex decreased the level of Histone H3 acetylation (H3ac) in the promoter regions of *PpRAP2.4* and *PpMYB114*, which inhibited the expression of these genes, and ultimately suppressed anthocyanin biosynthesis [52]. As DNA methylation and histone modifications have a dose effect, further study could be conducted to reveal the gradual changes in gene expression in response to leaf senescence.

### 2.5. Color Change in Different Tissues

Anthocyanin biosynthesis in fruits can improve the nutritional and commercial value of fruits. In the past few decades, the mechanisms regulating anthocyanin synthesis in the skin and flesh of fruits have been well understood. Core MYB transcription factors are regulated by upstream genes to activate or inhibit anthocyanin accumulation and promote or inhibit fruit coloring (Table 1). In addition, special mutant plants may be caused by the structural variation of a key gene in the anthocyanin biosynthesis pathway. For example, variation in the GhMYB113 gene directly caused the whole cotton to be brown, especially the cotton fiber, which is of significant economic importance [27].

Compared to fruits, leaves containing various polyphenols exhibit a much higher antioxidant capacity in blueberries and lingonberries [3]. Therefore, we hypothesize that anthocyanin accumulation in leaves plays a vital role, especially during senescence. As leaves age, their color markedly changes from green to yellow or red. This transformation occurs because trees cycle nutrients through their leaves, leading to the degradation of chlorophyl, as well as the appearance of carotenoids and other auxiliary photosynthetic pigments. The accumulation of anthocyanins provides a means to delay leaf senescence and helps plants adapt to environmental constraints [1]. Reports on anthocyanin biosynthesis in deciduous trees during leaf senescence showed that a class of MYB transcription factors are specifically expressed in the leaves [30,33]. In evergreen trees, leaf senescence is also associated with anthocyanin accumulation. *Cinnamomum camphora* is a material for extracting natural pigments due to its variety of leaf colors at different stages of maturity [53]. A study has shown that the significantly high expression of several bHLH genes in the bright red bark and leaves after half-lignification implied their role in anthocyanin biosynthesis [54]. Therefore, we believe that the accumulation of anthocyanin in senescent leaves is completely different from the process of fruit coloring.

In both deciduous trees and evergreen trees, leaf senescence is often accompanied by the process of anthocyanin biosynthesis (Table 2). The decline of photosynthetic capacity, the degradation of chlorophyll, and the accumulation of anthocyanins are the important signs of leaf senescence in *A. saccharum* [55]. WRKY and NAC TFs are often considered candidate genes to link anthocyanin biosynthesis to senescence, acting by activating MYB, which is specifically highly expressed in the senescence stage [32,56,57]. In *M. domestica*, *MdbHLH3* interacts with MdMYB1 enhancing anthocyanin content and fruit coloration, meanwhile regulating leaf senescence by directly increasing *MdDEP1* expression [58].

## 3. Factors Affecting Biosynthesis of Anthocyanin

### 3.1. Phytohormones

In addition to developmental age, leaf senescence is also influenced by numerous internal and external signals. Plant hormonal signals are combined with age information to regulate leaf senescence as major players [59]. Among them, ethylene, jasmonic acid, and abscisic acid act as primary inducers to promote this process [60,61,62]. These three phytohormones have been shown to play dominant roles in promoting leaf senescence. Here, we focus on these three kinds of hormones and their effects on anthocyanin biosynthesis during leaf senescence.

#### 3.1.1. Ethylene

Studies have shown that both plant ripening and senescence are sensitive to ethylene and are regulated by endogenous ethylene [63]. Ethylene plays dual roles in modulating anthocyanin accumulation in different plants. Its positive influence on fruit coloration has been demonstrated in a number of fruit crops, including grape, apple, and mulberry. In apples, ethylene accelerates anthocyanin accumulation by promoting the transcription of *MdMYB1* and other key genes in anthocyanin biosynthesis, while MdMYB1 induces the transcription of an *ETHYLENE RESPONSE FACTOR*, *MdERF3*, to further enhance ethylene-mediated anthocyanin accumulation and apple fruit coloration [64]. Ethylene treatment promotes the strong expression of *MaERF5*, which regulates anthocyanin biosynthesis in ‘Zijin’ mulberry (*Morus alba*) fruits by interacting with *MaMYBA* and *MaF3H* [65]. Conversely, *Arabidopsis* and pears serve as examples of how ethylene negatively affects the biosynthesis of anthocyanin. In *Arabidopsis*, ethylene suppresses anthocyanin accumulation by diminishing the expression of the anthocyanin activator *AtPAP1* and promoting the expression of anthocyanin repressor *AtMYBL2* [66]. PpERF105, activated by ethylene, stimulates the expression of the repressor-type transcription factor *PpMYB140*, which inhibits anthocyanin biosynthesis in red pear fruits [67].

**Figure 2:**
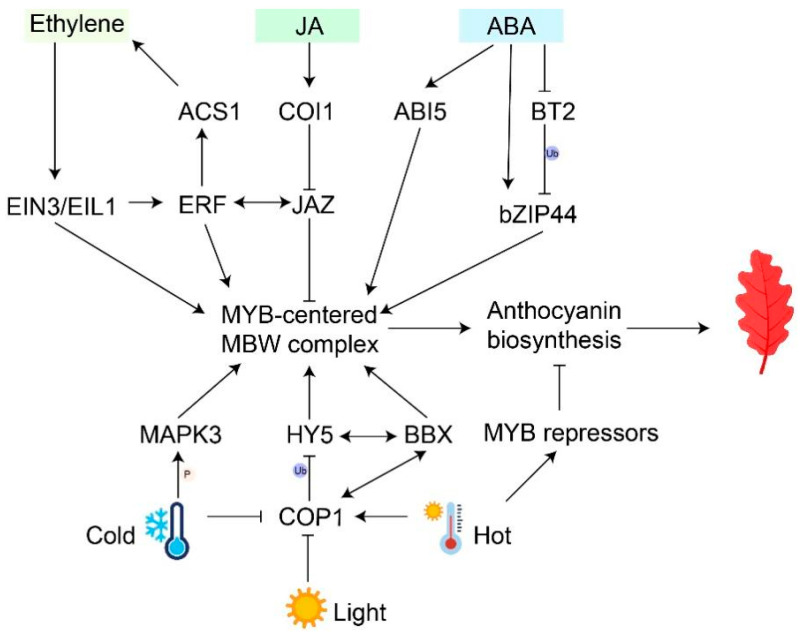
Regulatory pathways of environmental factors and phytohormones interactions in anthocyanin accumulation. Black arrows represent activations; ‘T’ arrows represent repressions; double-headed arrows represent interactions; ‘Ub’ represents ubiquitination; ‘P’ represents phosphorylation.

Considering the role of ethylene in leaf senescence, it can be speculated that ethylene plays a positive role in leaf coloration during senescence (Figure 2). Analysis of membrane transport proteins and hormone pathways in Arabidopsis during leaf growth showed that ACC synthetase (ACS) and ACC oxidase (ACO), encoded by ethylene biosynthesis genes, were up-regulated with leaf senescence, thus promoting ethylene accumulation [68]. After ethylene treatment, ETHYLENE INSENSITIVE3 (EIN3), a key transcription factor of ethylene signaling pathway, can directly activate the expression of master senescence-associated genes ORE1/NAC092 and SAG29 to accelerate chlorophyll degradation and leaf senescence [69,70]. In *Arabidopsis*, the *erf* mutant decreased the rate and extent of leaf anthocyanin production [71]. *MpERF105* and *MpNAC72*, induced by ethylene, positively regulates anthocyanin accumulation in fungal disease-infected *M.* ‘Profusion’ leaves by mediating the expression of *MpMYB10b* and enhancing rust resistance [72]. Ethylene signaling, which also promotes fruit coloration, regulates key genes in the anthocyanin biosynthesis pathway to adjust anthocyanin accumulation during leaf senescence.

#### 3.1.2. Abscisic Acid

Abscisic acid is crucial for both plant senescence and the promotion of anthocyanin biosynthesis. NCED is a key enzyme that promotes ABA biosynthesis. In strawberries, anthocyanin accumulation on the surface of *FaNCED1*-RNAi fruits was inhibited compared with control lines, and exogenous ABA treatment restored anthocyanin content in *FaNCED1*-RNAi fruits [73]. The mechanisms of ABA regulating the biosynthesis of anthocyanin have been clarified. ABA induces anthocyanin accumulation by activating MYB-centered MBW complex and improving the expression of structural genes (Figure 2). In sweet cherries, red pigment markedly enhanced, and the expression of anthocyanin activator *PacMYBA* significantly increased after ABA treatment [74]. Similarly, exogenous ABA treatment also induces the biosynthesis of anthocyanin via activating *FaMYB10* in strawberries [75]. In *Aristotelia chilensis*, the expression of *AcUFGT* decreased in fully-expanded leaves of stressed plants treated with fluridone, an inhibitor of ABA biosynthesis, while subsequent ABA application increased the *AcUFGT* expression [76]. Many TFs, like Basic Leucine Zipper (bZIP), have been reported to participate in the major ABA-dependent signaling pathways and act on downstream MYBs. In the absence of ABA, MdbZIP44 in apples was degraded and ubiquitinated by MdBT2, inhibiting fruit coloring. Under ABA treatment, ABA directly increased the expression of MdbZIP44 and inhibited MdBT2 expression, resulting in the release of the MdbZIP44 protein and improvement of anthocyanin accumulation [77]. Moreover, MdABI5 promotes anthocyanin accumulation by activating *MdbHLH3* and increasing the interaction between MdMYB1 and MdbHLH3 [78]. In the young leaves of the tea plant (*Camellia sinensis*), exogenous ABA induces the expression of *CsMYB4/44* and further activates transcription of bHLHs and MYBs, which directly activates anthocyanins’ biosynthesis and transport genes expression [79]. Given that ABA is accumulated during leaf senescence, it could be speculated that ABA promotes anthocyanin biosynthesis during leaf senescence, but the detailed mechanism needs further study.

#### 3.1.3. Jasmonic Acid

Jasmonic acid (JA) is a class of lipid plant hormones that plays important roles in plant defense and senescence [80]. JA signaling is perceived by the receptor COI1, and the JASMONATE ZIM-DOMAIN (JAZ) protein serves as a repressor in the JA signaling transduction pathway (Figure 2). The interaction of COI1 with JAZs leads to JAZ ubiquitination, resulting in the release of transcription factors and the activation of downstream gene expression [81]. The Arabidopsis JAZ proteins interact with bHLH (TT8) and MYB proteins (MYB75), reducing the transcriptional function of the MBW complex and inhibiting anthocyanin accumulation. Upon perception of JA signal, JAZ proteins are degraded, and the MBW complex is released to regulate anthocyanin biosynthesis [82]. Recent studies have shown that ECAP helps JAZ6/8 recruit TOPLESS-RELATED 2 (TPR2) to form a transcription suppressor complex in this process [83]. In addition, JA-induced degradation of MdJAZ5/10 leads to a high expression of MdMYC2 and MdMYB1/9/11, promoting anthocyanin accumulation in apples [84]. This result indicates that ethylene and jasmonic acid have a synergistic effect on the regulation of anthocyanin. During the leaf senescence of *P. chinensis*, bioactive jasmonic acid-isoleucine (JA-Ile) was markedly accumulated, and four JA signaling-related genes were reduced in the autumn leaf [85]. Overall, JA plays an important positive regulatory role in anthocyanin biosynthesis.

### 3.2. Environmental Factors

#### 3.2.1. Light

Light exposure has been proven to increase anthocyanin biosynthesis in plants (Figure 2) [23]. Without light, aging leaves could not accumulate anthocyanin [85]. Specifically, the quality, duration, and intensity of light have a significant impact on the accumulation of anthocyanins [17]. For instance, apple fruits directly exposed to light showed a more intense anthocyanin pigmentation compared to the wrapped ones [25]. In addition, light quality also affects anthocyanin biosynthesis [86,87]. UV-A irradiation and high red light have been reported to induce anthocyanin content in tomato seedlings compared to darkness [88,89]. Recently, the mechanism of light-controlled anthocyanin biosynthesis has been widely reported. CONSTITUTIVE PHOTOMORPHOGENIC1 (COP1) is a key suppressor in light signal transduction downstream of the photoreceptor. In darkness, MdCOP1 protein interacts with MdMYB1 and mediates its ubiquitination and degradation, thereby inhibiting apple fruit coloration [90]. Several transcription factors (e.g., HY5 and BBX) are involved in the light signal regulation of anthocyanin biosynthesis [39,91]. HY5 can not only directly activate structural genes but also regulate MYB transcription factors and the MBW complex to indirectly affect the expression of structural genes [92,93]. In purple pummelo (*Citrus grandis*), *CgHY5* is induced by light and directly binds to the G-box within an R2R3 MYB transcription factor (*CgRuby1*) promoter, leading to anthocyanin accumulation [94]. Recently, it has been reported that protein complexes regulate downstream gene expression by activating anthocyanin biosynthesis [95]. In poplars, PtrHY5 interacts with the PtrBBX23 gene through the C-terminal bZIP domain to enhance the expression of downstream genes and modulate the accumulation of anthocyanins in the leaf [96]. Some studies have shown that protein phosphorylation is a part of the light-induced developmental processes. Protein phosphorylation induced by mitogen-activated protein kinase (MAPK) contributes to anthocyanin accumulation [97]. Light-induced MPK4 phosphorylation of MYBs promotes its stability and increases anthocyanin accumulation in Arabidopsis and apples [98,99].

#### 3.2.2. Temperature

Temperature is also an important environmental factor that affects anthocyanin pigmentation during leaf senescence. Most studies have shown that high temperatures (HTs) restrain while low temperatures (LTs) induce anthocyanin accumulation (Figure 2) [16,100]. In physiological metabolism, LTs reduce the rate of dark respiration and accelerate the accumulation of sugar, further promoting anthocyanin biosynthesis [101]. At the transcriptional level, the *CsUGT75C1* gene is up-regulated, activating anthocyanin accumulation in *C. sinensis* leaves under LT conditions rather than HT [102]. HY5 could participate in and integrate low temperature and light signaling [91]. In Arabidopsis, LTs stimulate COP1 to become inactivated and be excluded from the nucleus, allowing HY5 stabilization and activation of anthocyanin biosynthesis genes [100]. In addition to directly activating structural genes, HY5 binds to either the G-box or ACE-box of MYB transcription factors to regulate anthocyanin biosynthesis [103,104,105]. With the deepening of research, MYB transcription factors are reported to specifically regulate LT-induced anthocyanin. The interaction between MdbHLH and MdMYB1 is enhanced, activating anthocyanin accumulation after exposure to LT [106]. The insertion of a low-temperature-responsive element (LTRE) in *CsRuby1* induced anthocyanin accumulation in pulp only at low temperatures [94]. Exceptionally, LTs lead to lower anthocyanin contents in strawberry fruit by stimulating the phosphorylation of FvMYB10 by MITOGEN-ACTIVATED PROTEIN KINASE 3 (FvMAPK3) [107]. Therefore, LTs might have dual roles in anthocyanin accumulation.

On the other hand, high temperatures would inhibit anthocyanin accumulation by reducing anthocyanin biosynthesis and promoting anthocyanin degradation. Several MYB repressors have been activated by HTs to reduce anthocyanin accumulation. CmMYB012 in chrysanthemum was induced and led to a decrease in anthocyanins by suppressing *CmCHS*, *CmDFR*, *CmANS*, and *CmUFGT* expressions [108]. HTs cause a reduction in anthocyanin biosynthesis in potatoes (*S. tuberosum*) by enhancing the expression of flesh-specific StMYB44 [109]. Additionally, anthocyanins are degraded by numerous enzymes during HTs. BcPrx01, a basic peroxidase, is responsible for the degradation of anthocyanins in *Brunfelsia calycina* flowers [110]. VviPrx31 peroxidase in grapes participates in anthocyanin degradation under high temperatures [111]. In summary, temperature is one of the key environmental factors affecting anthocyanin biosynthesis.

### 3.3. Nutrient Deficiency

Nutrients support growth and development, making them indispensable for the plant life cycle. Leaf senescence is characterized by the transition from nutrient assimilation to nutrient reactivation [112]. The earliest and most significant change in this process is the decomposition of chloroplasts. Chloroplasts are important sites for photosynthesis and storage of many nutrient elements [113]. Consequently, aging leaves of plants are confronted with nutrient deficiency and imbalances, which have been shown to be related to anthocyanin accumulation [114].

#### 3.3.1. Nitrogen Deficiency

**Figure 3:**
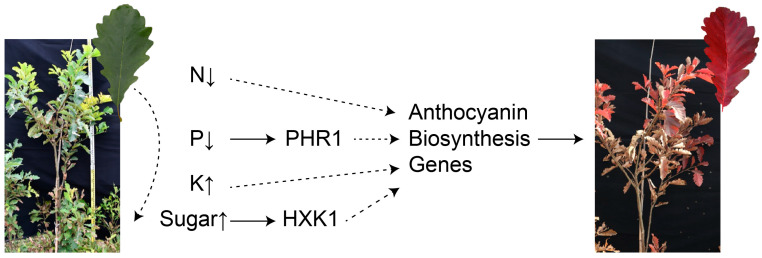
Changes in nutrients and effects on anthocyanin accumulation during leaf senescence. The curved dotted lines represent the downward transfer of nutrients from the leaves.

The nitrogen (N) content in senescent leaves significantly decreased [115]. Studies have demonstrated that anthocyanins accumulate in leaves when plants are grown under N deficiency conditions (Figure 3) [116,117]. During low N-induced leaf senescence, anthocyanin accumulations can minimize stress-related oxidative damage and facilitate nutrient remobilization from older leaves to younger active tissues to enhance their adaptation to low nitrogen [118]. In Arabidopsis, regulation of anthocyanin biosynthesis by the GA-DELLA module is important for plant adaptation to a nitrogen deficiency [119]. This deficiency of N increases anthocyanin biosynthesis by up-regulating the expression of structural genes such as *PAL*, *CHS*, and *F3H* in tomatoes [120]. In *M. spectabilis*, the total anthocyanin content and cyanidin-3-O-galactoside chloride in the explants accumulate obviously under low-nitrogen conditions [43,121]. As major forms of N, nitrate (NO_3_^−^) and ammonium (NH_4_^+^) regulate plant growth as signals [122]. Media generally contain NO_3_^−^ and ammonium NH_4_^+^ ions as nitrogen sources and significantly influence the growth and metabolism of plant tissue. In many plants, including *Catharanthus roseus*, *Cleome rosea*, and *A. thaliana*, a decrease in NO_3_^−^ and NH_4_^+^ levels leads to a greater accumulation of anthocyanins [123,124,125]. This suggests that plant responses to nitrogen concentrations can protect sensitive plants from stress by inducing more anthocyanins. This hypothesis has been verified in *A. thaliana*: under low nitrate conditions, the *PAP1-D/fls1ko* mutants with significant anthocyanin accumulation show higher salt tolerance than the *ttg1* anthocyanin-deficient mutants [126]. Additionally, sucrose is involved in low nitrogen-induced anthocyanin accumulation. Increased sucrose in the hypocotyls of radish sprouts contributes to nitrogen deficiency-induced anthocyanin accumulation [127].

#### 3.3.2. Phosphorus Deficiency

Phosphorus (P), like nitrogen, decreased significantly in senescent leaves (Figure 3) [115]. As a general plant response, an increase in the anthocyanin content occurs under P deficiency, as reported for multiple plant species, such as *A. thaliana*, *Zea mays*, *S. lycopersicum*, and *Triticum aestivum* [128,129,130,131,132]. Total anthocyanin accumulation was observed in suspension-cultured grape (*Vitis vinifera*) cells in vitro under P deficiency [133]. In general, foliar anthocyanin production is associated with P deficiency to enhance plant tolerance [134]. These results reveal that anthocyanins act as defense substances that help plants adapt to a nutrient deficiency. Like nitrogen deficiency, deficiency of P increases anthocyanin biosynthesis by regulating structural genes and transcription factors. PHOSPHATE STARVATION RESPONSE1 (PHR1) plays key roles in P deficiency-induced anthocyanin biosynthesis in plants. MdPHR1 is activated by P-deficient stress and interacts with MdWRKY75 to enhance the transcription of *MdMYB1*, leading to anthocyanin biosynthesis in apples [135]. Similarly, in Arabidopsis, the *DFR* gene is increased in *phr1* mutants, and anthocyanin accumulates under the condition of P deficiency [136].

#### 3.3.3. Potassium

Potassium, as an essential nutrient, plays an important role in anthocyanin biosynthesis. Research shows that low K could significantly increase the content of soluble sugar and anthocyanins in the skin of grapes [137]. Similarly, this conclusion has been confirmed in litchis [138]. Potassium appears to facilitate anthocyanin synthesis by enhancing the accumulation and transport of sugars.

#### 3.3.4. Changes in Sugars

The level of sugars regulates plant growth and development. Extensive evidence shows that carbohydrate storage products in leaves reduce photosynthetic activity and induce leaf senescence [139,140,141]. For example, there are higher sugar levels in tobacco leaves at the edge of senescence than in younger or older tobacco leaves [112]. In addition, sugars induce anthocyanin biosynthesis in various plant species. They not only provide carbon sources, skeletons, and glucosides for anthocyanin biosynthesis but also increase the expression levels of biosynthetic structural genes and regulatory *MYB* genes [142,143,144]. PRODUCTION OF ANTHOCYANIN PIGMENT1 (PAP1) in Arabidopsis, the major TF regulating anthocyanin biosynthesis, responds positively to increased sucrose concentrations [142]. In our research, it was also found that 30 g/L of sucrose could significantly promote the leaf coloring of *P. chinensis* in autumn [145]. With further research, Hexokinase 1 (HXK1) was considered to be a sugar sensor in plants and crosstalk with ABA, ethylene, auxin, cytokinin, and brassinosteroid signaling [145,146]. In the presence of glucose, MdHXK1 protein kinase stabilizes MdbHLH3 by phosphorylation to increase anthocyanin accumulation in apples [147]. Therefore, we hypothesized that senescent leaves induce hormone crosstalk through sugar accumulation and promote the expression of anthocyanin genes to increase leaf coloring (Figure 3).

## 4. Conclusions and Perspectives

Anthocyanin accumulation during leaf senescence is an essential process in response to biological and abiotic stresses. Despite substantial advances in the understanding of the regulatory mechanism of anthocyanin pigmentation in fruit over the last few decades, the study of leaf coloration during senescence remains insufficient. Our current consensus is that a low temperature may be the main environmental factor that promotes leaf coloration in autumn, especially for deciduous trees. We propose a regulatory model suggesting that changes in the environment lead to the accumulation of ethylene, ABA, JA, and other hormones, resulting in the differential expression of transcription factors in their signal pathway. These TFs interact with core MYB transcription factors, thereby upregulating structural genes in the anthocyanin biosynthesis pathway. As a conspicuous aspect of leaf senescence, anthocyanin accumulation is typically accompanied by chlorophyll degradation. It has been confirmed that some transcription factors (e.g., NAC) can simultaneously activate key genes in chlorophyll degradation and anthocyanin biosynthesis, thereby initially linking the two processes. However, several major issues related to anthocyanin accumulation in leaf coloration need to be addressed in the future:

(1) What are the differences in anthocyanin biosynthesis mechanisms at different leaf stages, such as young leaves and old leaves? What are the key transcription factors specifically regulating anthocyanin biosynthesis in response to leaf senescence? Answers to these questions will contribute to the molecular breeding of ornamental plants with different colors in different seasons.
(2) What is the transcriptional regulatory network between chlorophyll degradation and anthocyanin biosynthesis? How can anthocyanin biosynthesis be promoted, which delays leaf senescence at the same time? Addressing these questions will help extend the ornamental period of the plants.
(3) How can leaf coloration be facilitated through artificial intervention under abnormal temperature conditions in nature for landscaping purposes?

## Figures and Tables

**Table 1:** Color changes in different tissues.

Specie	Tissue	Transcription Factor Regulation	Changes in Anthocyanin	Year
*Pyrus pvrifolia*	Peel	BBX16 induced MYB10 expression	Increase	2019
*Pyrus pvrifolia*	Peel	BBX18 and BBX21 antagonistically regulate MYB10 expression		2019
*Prunus persica*	Peel	MYB18 competed with MYB10	Decrease	2018
*Malus domestica*	Peel	EIL1 enhanced MYB1 action	Increase	2018
*Fragaria vesca* *Fragaria* × *ananassa*	Skin and flesh	MYB10	Increase	2020
*Actinidia chinensis*	Pulp	MYB10 and MYB110	Increase	2022
*Mimulus*	Flower	PELAN	Inhibition	2023
*Pyrus pvrifolia*	Whole plant	BBX24	Increase	2020
*Gossypium hirsutum*	Whole plant	MYB113	Increase	2022
*Quercus dentata*	Autumn leaf	MYB	Increase	2023
*Quercus aliena*	Autumn leaf	MYB1 and MYB3	Increase	2022
*Pistacia chinensis*	Autumn leaf	MYB113	Increase	2021
*Liquidambar formosana*	Autumn leaf Young leaf	MYB5 and MYB123 increased in spring; MYB113 increased in late autumn	Increase	2021
*Cinnamomum camphora*	Bark and leaf	Several bHLH genes	Increase	2023

**Table 2:** Regulations of anthocyanin during leaf senescence.

Specie	Classification	Key Genes	Changes in Anthocyanin	Year
*Acer saccharum*	Deciduous tree		Increase	2016
*Liquidambar formosana*	Deciduous tree	WRKY75, NAC1 and MYB113	Increase	2015
*Liquidambar formosana*	Deciduous tree	MYB113	Increase	2021
*Malus domestica*	Deciduous tree	bHLH3-MYB1; bHLH3-DEP1	Increase	2020
*Malus spectabilis*	Deciduous tree	eTM-miR858-MYB62-like module	Increase	2023
*Pistacia chinensis*	Deciduous tree	JA signaling-related genes	Increase	2021
*Pistacia chinensis*	Deciduous tree	MYB113	Increase	2021
*Prunus persica*	Deciduous tree	NAC1-MYB10.1	Increase	2023
*Quercus aliena*	Deciduous tree	MYB1 and MYB3	Increase	2022
*Quercus dentata*	Deciduous tree	NAC, MYB	Increase	2023
*Cinnamomum camphora*	Evergreen tree	bHLH genes	Increase	2023

## Data Availability

The study did not report any data.

## References

[1] Landi M., Tattini M., Gould K.S. (2015). Multiple functional roles of anthocyanins in plant-environment interactions. Environ. Exp. Bot..

[2] Bendokas V., Skemiene K., Trumbeckaite S., Stanys V., Passamonti S., Borutaite V., Liobikas J. (2020). Anthocyanins: From plant pigments to health benefits at mitochondrial level. Crit. Rev. Food Sci. Nutr..

[3] Kelly E., Vyas P., Weber J.T. (2018). Biochemical Properties and Neuroprotective Effects of Compounds in Various Species of Berries. Molecules.

[4] Cabrita L., Fossen T., Andersen O.M. (2000). Colour and stability of the six common anthocyanidin 3-glucosides in aqueous solutions. Food Chem..

[5] Tanaka Y., Sasaki N., Ohmiya A. (2008). Biosynthesis of plant pigments: Anthocyanins, betalains and carotenoids. Plant J..

[6] Shoji K., Miki N., Nakajima N., Momonoi K., Kato C., Yoshida K. (2007). Perianth bottom-specific blue color development in Tulip cv. Murasakizuisho requires ferric ions. Plant Cell Physiol..

[7] Yoshida K., Toyama-Kato Y., Kameda K., Kondo T. (2003). Sepal Color Variation of Hydrangea macrophylla and Vacuolar pH Measured with a Proton-Selective Microelectrode. Plant Cell Physiol..

[8] Sousa A., Araújo P., Azevedo J., Cruz L., Fernandeset I., Mateus N., de Freitas V. (2016). Antioxidant and antiproliferative properties of 3-deoxyanthocyanidins. Food Chem..

[9] Masci A., Coccia A., Lendaro E., Mosca L., Paolicelli P., Cesa S. (2016). Evaluation of different extraction methods from pomegranate whole fruit or peels and the antioxidant and antiproliferative activity of the polyphenolic fraction. Food Chem..

[10] Gonçalves A., Rodrigues M., Santos A., Alves G., Silva L. (2018). Antioxidant Status, Antidiabetic Properties and Effects on Caco-2 Cells of Colored and Non-Colored Enriched Extracts of Sweet Cherry Fruits. Nutrients.

[11] Lage N.N., Layosa M.A.A., Arbizu S., Chew B.P., Pedrosa M.L., Mertens-Talcott S., Talcott S., Noratto G.D. (2020). Dark sweet cherry (*Prunus avium*) phenolics enriched in anthocyanins exhibit enhanced activity against the most aggressive breast cancer subtypes without toxicity to normal breast cells. J. Funct. Foods.

[12] Forester S.C., Choy Y.Y., Waterhouse A.L., Oteiza P.I. (2014). The anthocyanin metabolites gallic acid, 3-O-methylgallic acid, and 2,4,6-trihydroxybenzaldehyde decrease human colon cancer cell viability by regulating pro-oncogenic signals. Mol. Carcinog..

[13] Chen L., Teng H., Fang T., Xiao J.B. (2016). Agrimonolide from Agrimonia pilosa suppresses inflammatory responses through down-regulation of COX-2/iNOS and inactivation of NF-κB in lipopolysaccharide-stimulated macrophages. Phytomedicine.

[14] Wang Y., Zhang D., Liu Y.X., Wang D., Liu J., Ji B.P. (2015). The protective effects of berry-derived anthocyanins against visible light-induced damage in human retinal pigment epithelial cells. J. Sci. Food Agric..

[15] Holton T.A., Cornish E.C. (1995). Genetics and Biochemistry of Anthocyanin Biosynthesis. Plant Cell.

[16] Lin-Wang K., Micheletti D., Palmer J., Volz R., Lozano L., Espley R., Hellens R.P., Chagne D., Rowan D.D., Troggio M. (2011). High temperature reduces apple fruit colour via modulation of the anthocyanin regulatory complex. Plant Cell Environ..

[17] Jaakola L. (2013). New insights into the regulation of anthocyanin biosynthesis in fruits. Trends Plant Sci..

[18] Wang X., Chen X.P., Luo S.X., Ma W., Li N., Zhang W.W., Tikunov Y., Xuan S.X., Zhao J.J., Wang Y.H. (2022). Discovery of a DFR gene that controls anthocyanin accumulation in the spiny Solanum group: Roles of a natural promoter variant and alternative splicing. Plant J..

[19] Lu Z.H., Cao H.H., Pan L., Niu L., Wei B., Cui G.C., Wang L.W., Yao J.L., Zeng W.F., Wang Z.Q. (2021). Two loss-of-function alleles of the glutathione S-transferase (GST) gene cause anthocyanin deficiency in flower and fruit skin of peach (*Prunus persica*). Plant J..

[20] Xu W.J., Dubos C., Lepiniec L. (2015). Transcriptional control of flavonoid biosynthesis by MYB-bHLH-WDR complexes. Trends Plant Sci..

[21] Feller A., Machemer K., Braun E.L., Grotewold E. (2011). Evolutionary and comparative analysis of MYB and bHLH plant transcription factors. Plant J..

[22] Wang W.Q., Moss S.M.A., Zeng L.H., Espley R.V., Wang T.C., Lin-Wang K., Fu B.L., Schwinn K.E., Allan A.C., Yin X.R. (2022). The red flesh of kiwifruit is differentially controlled by specific activation-repression systems. New Phytol..

[23] Takos A.M., Jaffe F.W., Jacob S.R., Bogs J., Robinson S.P., Walker A.R. (2006). Light-Induced Expression of a MYB Gene Regulates Anthocyanin Biosynthesis in Red Apples. Plant Physiol..

[24] Ban Y., Honda C., Hatsuyama Y., Igarashi M., Bessho H., Moriguchi T. (2007). Isolation and Functional Analysis of a MYB Transcription Factor Gene that is a Key Regulator for the Development of Red Coloration in Apple Skin. Plant Cell Physiol..

[25] Hu Y.J., Cheng H., Zhang Y., Zhang J., Niu S.Q., Wang X.S., Li W.J., Zhang J., Yao Y.C. (2021). The MdMYB16/MdMYB1-miR7125-MdCCR module regulates the homeostasis between anthocyanin and lignin biosynthesis during light induction in apple. New Phytol..

[26] Castillejo C., Waurich V., Wagner H., Ramos R., Oiza N., Munoz P., Trivino J.C., Caruana J., Liu Z.C., Cobo N. (2020). Allelic Variation of MYB10 Is the Major Force Controlling Natural Variation in Skin and Flesh Color in Strawberry (*Fragaria* spp.). Fruit. Plant Cell.

[27] Wang N., Zhang B.B., Yao T., Shen C., Wen T.W., Zhang R.T., Li Y.X., Le Y., Li Z.H., Zhang X.L. (2022). Re enhances anthocyanin and proanthocyanidin accumulation to produce red foliated cotton and brown fiber. Plant Physiol..

[28] Liang M., Chen W.J., LaFountain A.M., Liu Y.L., Peng F., Xia R., Bradshaw H.D., Yuan Y.-W. (2023). Taxon-specific, phased siRNAs underlie a speciation locus in monkeyflowers. Science.

[29] Sun S.J., Zhang Q., Yu Y.F., Feng J.Y., Liu C.L., Yang J.D. (2022). Leaf Coloration in *Acer palmatum* Is Associated with a Positive Regulator ApMYB1 with Potential for Breeding Color-Leafed Plants. Plants.

[30] Song X.H., Yang Q.S., Liu Y., Li J.J., Chang X.C., Xian L.H., Zhang J. (2021). Genome-wide identification of Pistacia R2R3-MYB gene family and function characterization of PcMYB113 during autumn leaf coloration in *Pistacia chinensis*. Int. J. Biol. Macromol..

[31] Yang X., Yang N., Zhang Q., Pei Z.Q., Chang M.X., Zhou H.R., Ge Y.Y., Yang Q.S., Li G.L. (2022). Anthocyanin Biosynthesis Associated with Natural Variation in Autumn Leaf Coloration in *Quercus aliena* Accessions. Int. J. Mol. Sci..

[32] Wang W.B., He X.F., Yan X.M., Lu F.C., Wu J., Zheng Y., Wang W.H., Xue W.B., Tian X.C., Guo J.F. (2023). Chromosome-scale genome assembly and insights into the metabolome and gene regulation of leaf color transition in an important oak species, *Quercus dentata*. New Phytol..

[33] Wen C.H., Tsao N.W., Wang S.Y., Chu F.H. (2021). Color variation in young and senescent leaves of Formosan sweet gum (*Liquidambar formosana*) by the gene regulation of anthocyanidin biosynthesis. Physiol. Plant..

[34] Ma D.W., Constabel C.P. (2019). MYB Repressors as Regulators of Phenylpropanoid Metabolism in Plants. Trends Plant Sci..

[35] Yin X.J., Zhang Y.B., Zhang L., Wang B.H., Zhao Y.D., Irfan M., Chen L.J., Feng Y.L. (2021). Regulation of MYB Transcription Factors of Anthocyanin Synthesis in Lily Flowers. Front. Plant Sci..

[36] Zhou H., Lin-Wang K., Wang F., Espley R.V., Ren F., Zhao J.B., Ogutu C., He H.P., Jiang Q., Allan A.C. (2018). Activator-type R2R3-MYB genes induce a repressor-type R2R3-MYB gene to balance anthocyanin and proanthocyanidin accumulation. New Phytol..

[37] Wei Z.Z., Hu K.D., Zhao D.L., Tang J., Huang Z.Q., Jin P., Li Y.H., Han Z., Hu L.Y., Yao G.F. (2020). MYB44 competitively inhibits the formation of the MYB340-bHLH2-NAC56 complex to regulate anthocyanin biosynthesis in purple-fleshed sweet potato. BMC Plant Biol..

[38] Li X.J., Martin-Pizarro C., Zhou L.L., Hou B.Z., Wang Y.Y., Shen Y.Y., Li B.B., Pose D., Qin G.Z. (2023). Deciphering the regulatory network of the NAC transcription factor FvRIF, a key regulator of strawberry (*Fragaria vesca*) fruit ripening. Plant Cell.

[39] Bai S.L., Tao R.Y., Tang Y.X., Yin L., Ma Y.J., Ni J.B., Yan X.H., Yang Q.S., Wu Z.Y., Zeng Y.L. (2019). BBX16, a B-box protein, positively regulates light-induced anthocyanin accumulation by activatingMYB10in red pear. Plant Biotechnol. J..

[40] Bai S.L., Tao R.Y., Yin L., Ni J.B., Yang Q.S., Yan X.H., Yang F., Guo X.P., Li H.X., Teng Y.W. (2019). Two B-box proteins, PpBBX18 and PpBBX21, antagonistically regulate anthocyanin biosynthesis via competitive association with *Pyrus pyrifolia* ELONGATED HYPOCOTYL 5 in the peel of pear fruit. Plant J..

[41] Yang G.Y., Sun M.Y., Brewer L., Tang Z.K., Nieuwenhuizen N., Cooney J., Xu S.Z., Sheng J.W., Andre C., Xue C. (2024). Allelic variation of BBX24 is a dominant determinant controlling red coloration and dwarfism in pear. Plant Biotechnol. J..

[42] Su M.Y., Zuo W.F., Wang Y.C., Liu W.J., Zhang Z.Y., Wang N., Chen X.S. (2022). The WKRY transcription factor MdWRKY75 regulates anthocyanins accumulation in apples (*Malus domestica*). Funct. Plant Biol..

[43] Meng J.R., Sun S.H., Li A., Pan L., Duan W.Y., Cui G.C., Xu J., Niu L., Wang Z.Q., Zeng W.F. (2023). The eTM-miR858-MYB62-like module regulates anthocyanin biosynthesis under low-nitrogen conditions in *Malus spectabilis*. New Phytol..

[44] Gou J.Y., Felippes F.F., Liu C.J., Weigel D., Wang J.W. (2011). Negative Regulation of Anthocyanin Biosynthesis in Arabidopsis by a miR156-Targeted SPL Transcription Factor. Plant Cell.

[45] Deng J., Fu Z.Y., Chen S., Damaris R.N., Wang K., Li T.T., Yang P.F. (2015). Proteomic and Epigenetic Analyses of Lotus (*Nelumbo nucifera*) Petals Between Red and White cultivars. Plant Cell Physiol..

[46] Wang Z.G., Meng D., Wang A.D., Li T.L., Jiang S.L., Cong P.H., Li T.Z. (2013). The methylation of the *PcMYB10* promoter is associated with green-skinned sport in Max Red Bartlett pear. Plant Physiol..

[47] El-Sharkawy I., Liang D., Xu K. (2015). Transcriptome analysis of an apple (*Malus* × *domestica*) yellow fruit somatic mutation identifies a gene network module highly associated with anthocyanin and epigenetic regulation. J. Exp. Bot..

[48] Bai S.L., Tuan P.A., Saito T., Honda C., Hatsuyama Y., Ito A., Moriguchi T. (2016). Epigenetic regulation of *MdMYB1* is associated with paper bagging-induced red pigmentation of apples. Planta.

[49] Yu L.J., Sun Y.Y., Zhang X., Chen M.C., Wu T., Zhang J., Xing Y.F., Tian J., Yao Y.C. (2022). ROS1 promotes low temperature-induced anthocyanin accumulation in apple by demethylating the promoter of anthocyanin-associated genes. Hortic. Res..

[50] Cai H.Y., Zhang M., Chai M.N., He Q., Huang X.Y., Zhao L.H., Qin Y. (2019). Epigenetic regulation of anthocyanin biosynthesis by an antagonistic interaction between H2A.Z and H3K4me3. New Phytol..

[51] Fan D., Wang X.Q., Tang X.F., Ye X., Ren S., Wang D.H., Luo K.M. (2018). Histone H3K9 demethylase JMJ25 epigenetically modulates anthocyanin biosynthesis in poplar. Plant J..

[52] Ni J.B., Wang S.M., Yu W.J., Liao Y.F., Pan C., Zhang M.M., Tao R.Y., Wei J., Gao Y.H., Wang D.S. (2023). The ethylene-responsive transcription factor PpERF9 represses *PpRAP2.4* and *PpMYB114* via histone deacetylation to inhibit anthocyanin biosynthesis in pear. Plant Cell.

[53] Gong K., Pan Y., Rather L.J., Wang W.C., Zhou Q., Zhang T.H., Li Q. (2019). Natural pigment during flora leaf senescence and its application in dyeing and UV protection finish of silk and wool—A case study of Cinnamomum Camphora. Dyes Pigment..

[54] Gong X., Shen T.F., Li X.Q., Lin H.B., Chen C.H., Li H.H., Wu Z.X., Liu Q.L., Xu M., Zhang B. (2023). Genome-Wide Characterization and Analysis of bHLH Transcription Factors Related to Anthocyanin Biosynthesis *in Cinnamomum camphora* (‘Gantong 1’). Int. J. Mol. Sci..

[55] Junker L.V., Ensminger I. (2016). Relationship between leaf optical properties, chlorophyll fluorescence and pigment changes in senescing *Acer saccharum* leaves. Tree Physiol..

[56] Meng J.R., Sun S.H., Li A., Pan L., Duan W.Y., Cui G.C., Xu J., Niu L., Wang Z.Q., Zeng W.F. (2023). A NAC transcription factor, PpNAC1, regulates the expression of *PpMYB10.1* to promote anthocyanin biosynthesis in the leaves of peach trees in autumn. Hortic. Adv..

[57] Wen C.H., Lin S.S., Chu F.H. (2015). Transcriptome Analysis of a Subtropical Deciduous Tree: Autumn Leaf Senescence Gene Expression Profile of Formosan Gum. Plant Cell Physiol..

[58] Hu D.G., Sun C.H., Zhang Q.Y., Gu K.D., Hao Y.J. (2020). The basic helix-loop-helix transcription factor MdbHLH3 modulates leaf senescence in apple via the regulation of *dehydratase-enolase-phosphatase complex 1*. Hortic. Res..

[59] Woo H.R., Kim H.J., Lim P.O., Nam H.G. (2019). Leaf Senescence: Systems and Dynamics Aspects. Annu. Rev. Plant Biol..

[60] Gan S., Amasino R.M. (1997). Making sense of senescence (molecular genetic regulation and manipulation of leaf senescence). Plant Physiol..

[61] Lim P.O., Kim H.J., Nam H.G. (2007). Leaf Senescence. Annu. Rev. Plant Biol..

[62] Zhang Y., Tan S.Y., Gao Y.H., Kan C.C., Wang H.L., Yang Q., Xia X.L., Ishida T., Sawa S., Guo H.W. (2022). CLE42 delays leaf senescence by antagonizing ethylene pathway in *Arabidopsis*. New Phytol..

[63] Van Doorn W.G. (2002). Effect of Ethylene on Flower Abscission: A Survey. Ann. Bot..

[64] An J.P., Wang X.F., Li Y.Y., Song L.Q., Zhao L.L., You C.X., Hao Y.J. (2018). EIN3-LIKE1, MYB1, and ETHYLENE RESPONSE FACTOR3 Act in a Regulatory Loop That Synergistically Modulates Ethylene Biosynthesis and Anthocyanin Accumulation. Plant Physiol..

[65] Mo R.L., Han G.M., Zhu Z.X., Essemine J., Dong Z.X., Li Y., Deng W., Qu M.N., Zhang C., Yu C. (2022). The Ethylene Response Factor ERF5 Regulates Anthocyanin Biosynthesis in ‘Zijin’ Mulberry Fruits by Interacting with *MYBA* and *F3H* Genes. Int. J. Mol. Sci..

[66] Jeong S.W., Das P.K., Jeoung S.C., Song J.Y., Lee H.K., Kim Y.K., Kim W.J., Park Y.I., Yoo S.D., Choi S.B. (2010). Ethylene suppression of sugar-induced anthocyanin pigmentation in Arabidopsis. Plant Physiol..

[67] Ni J.B., Premathilake A.T., Gao Y.H., Yu W.J., Tao R.Y., Teng Y.W., Bai S.L. (2021). Ethylene-activated PpERF105 induces the expression of the repressor-type R2R3-MYB gene *PpMYB140* to inhibit anthocyanin biosynthesis in red pear fruit. Plant J..

[68] Graaff E.v.d., Schwacke R., Schneider A., Desimone M., Flugge U.I., Kunze R. (2006). Transcription Analysis of Arabidopsis Membrane Transporters and Hormone Pathways during Developmental and Induced Leaf Senescence. Plant Physiol..

[69] Qiu K., Li Z.P., Yang Z., Chen J.Y., Wu S.X., Zhu X.Y., Gao S., Gao J., Ren G.D., Kuai B.K. (2015). EIN3 and ORE1 Accelerate Degreening during Ethylene-Mediated Leaf Senescence by Directly Activating Chlorophyll Catabolic Genes in *Arabidopsis*. PLoS Genet..

[70] Wang C.Q., Dai S.Y., Zhang Z.L., Lao W.Q., Wang R.Y., Meng X.Q., Zhou X. (2021). Ethylene and salicylic acid synergistically accelerate leaf senescence in *Arabidopsis*. J. Integr. Plant Biol..

[71] Koyama T., Sato F. (2018). The function of ETHYLENE RESPONSE FACTOR genes in the light-induced anthocyanin production of *Arabidopsis thaliana* leaves. Plant Biotechnol..

[72] Wang Y., An H., Yang Y., Yi C., Duan Y., Wang Q., Guo Y.N., Yao L.N., Chen M.K., Meng J.X. (2024). The MpNAC72/MpERF105-MpMYB10b module regulates anthocyanin biosynthesis in *Malus* ‘Profusion’ leaves infected with *Gymnosporangium Yamadae*. Plant J..

[73] Jia H.F., Chai Y.M., Li C.L., Lu D., Luo J.J., Qin L., Shen Y.Y. (2011). Abscisic acid plays an important role in the regulation of strawberry fruit ripening. Plant Physiol..

[74] Shen X.J., Zhao K., Liu L.L., Zhang K.C., Yuan H.Z., Liao X., Wang Q., Guo X.W., Li F., Li T.H. (2014). A Role for PacMYBA in ABA-Regulated Anthocyanin Biosynthesis in Red-Colored Sweet Cherry cv. Hong Deng (*Prunus avium* L.). Plant Cell Physiol..

[75] Kadomura-Ishikawa Y., Miyawaki K., Takahashi A., Masuda T., Noji S. (2014). Light and abscisic acid independently regulated *FaMYB10* in *Fragaria* × *ananassa* fruit. Planta.

[76] González-Villagra J., Cohen J.D., Reyes-Díaz M.M. (2018). Abscisic acid is involved in phenolic compounds biosynthesis, mainly anthocyanins, in leaves of *Aristotelia chilensis* plants (Mol.) subjected to drought stress. Physiol. Plant..

[77] An J.P., Yao J.F., Xu R.R., You C.X., Wang X.F., Hao Y.J. (2018). Apple bZIP transcription factor MdbZIP44 regulates abscisic acid-promoted anthocyanin accumulation. Plant Cell Environ..

[78] An J.P., Zhang X.W., Liu Y.J., Wang X.F., You C.X., Hao Y.J. (2021). ABI5 regulates ABA-induced anthocyanin biosynthesis by modulating the MYB1-bHLH3 complex in apple. J. Exp. Bot..

[79] Gao C.X., Sun Y., Li J., Zhou Z., Deng X.M., Wang Z.H., Wu S.L., Lin L., Huang Y., Zeng W. (2023). High Light Intensity Triggered Abscisic Acid Biosynthesis Mediates Anthocyanin Accumulation in Young Leaves of Tea Plant (*Camellia sinensis*). Antioxidants.

[80] Griffiths G. (2020). Jasmonates: Biosynthesis, perception and signal transduction. Essays Biochem..

[81] Xu D.B., Ma Y.N., Qin T.F., Tang W.L., Qi X.W., Wang X., Liu R.C., Fang H.L., Chen Z.Q., Liang C.Y. (2021). Transcriptome-Wide Identification and Characterization of the JAZ Gene Family in *Mentha canadensis* L.. Int. J. Mol. Sci..

[82] Qi T.C., Song S.S., Ren Q.C., Wu D.W., Huang H., Chen Y., Fan M., Peng W., Ren C.M., Xie D.X. (2011). The Jasmonate-ZIM-Domain Proteins Interact with the WD-Repeat/bHLH/MYB Complexes to Regulate Jasmonate-Mediated Anthocyanin Accumulation and Trichome Initiation in *Arabidopsis thaliana*. Plant Cell.

[83] Li C.J., Shi L., Wang Y.N., Li W., Chen B.Q., Zhu L., Fu Y. (2020). *Arabidopsis* ECAP Is a New Adaptor Protein that Connects JAZ Repressors with the TPR2 Co-repressor to Suppress Jasmonate-Responsive Anthocyanin Accumulation. Mol. Plant.

[84] Wang S., Li L.X., Fang Y., Li D., Mao Z.L., Zhu Z.H., Chen X.S., Feng S.Q. (2022). MdERF1B–MdMYC2 module integrates ethylene and jasmonic acid to regulate the biosynthesis of anthocyanin in apple. Hortic. Res..

[85] Song X.H., Duan X.J., Chang X.C., Xian L.H., Yang Q.S., Liu Y. (2021). Molecular and metabolic insights into anthocyanin biosynthesis during leaf coloration in autumn. Environ. Exp. Bot..

[86] Tao R.Y., Bai S.L., Ni J.B., Yang Q.S., Zhao Y., Teng Y.W. (2018). The blue light signal transduction pathway is involved in anthocyanin accumulation in ‘Red Zaosu’ pear. Planta.

[87] Ni J.B., Liao Y.F., Zhang M.M., Pan C., Yang Q.S., Bai S.L., Teng Y.W. (2022). Blue Light Simultaneously Induces Peel Anthocyanin Biosynthesis and Flesh Carotenoid/Sucrose Biosynthesis in Mango Fruit. J. Agric. Food Chem..

[88] Fang H.C., Dong Y.H., Yue X.X., Chen X.L., He N.B., Hu J.F., Jiang S.H., Xu H.F., Wang Y.C., Su M.Y. (2019). MdCOL4 Interaction Mediates Crosstalk Between UV-B and High Temperature to Control Fruit Coloration in Apple. Plant Cell Physiol..

[89] Zhou L.J., Wang Y.X., Wang Y.G., Song A.P., Jiang J.F., Chen S.M., Ding B.Q., Guan Z.Y., Chen F.D. (2022). Transcription factor CmbHLH16 regulates petal anthocyanin homeostasis under different lights in Chrysanthemum. Plant Physiol..

[90] Li Y.Y., Mao K., Zhao C., Zhao X.Y., Zhang H.L., Shu H.R., Hao Y.J. (2012). MdCOP1 Ubiquitin E3 Ligases Interact with MdMYB1 to Regulate Light-Induced Anthocyanin Biosynthesis and Red Fruit Coloration in Apple. Plant Physiol..

[91] Gangappa S.N., Botto J.F. (2016). The Multifaceted Roles of HY5 in Plant Growth and Development. Mol. Plant.

[92] An J.P., Qu F.J., Yao J.F., Wang X.N., You C.X., Wang X.F., Hao Y.J. (2017). The bZIP transcription factor MdHY5 regulates anthocyanin accumulation and nitrate assimilation in apple. Hortic. Res..

[93] Wang Y.L., Wang Y.Q., Song Z.Q., Zhang H.Y. (2016). Repression of MYBL2 by Both microRNA858a and HY5 Leads to the Activation of Anthocyanin Biosynthetic Pathway in *Arabidopsis*. Mol. Plant.

[94] Huang D., Yuan Y., Tang Z.Z., Huang Y., Kang C.Y., Deng X.X., Xu Q. (2019). Retrotransposon promoter of Ruby1 controls both light- and cold-induced accumulation of anthocyanins in blood orange. Plant Cell Environ..

[95] Xu D.Q. (2019). COP1 and BBXs-HY5-mediated light signal transduction in plants. New Phytol..

[96] Li C.F., Pei J.L., Yan X., Cui X., Tsuruta M., Liu Y., Lian C.L. (2021). A poplar B-box protein PtrBBX23 modulates the accumulation of anthocyanins and proanthocyanidins in response to high light. Plant Cell Environ..

[97] Xing Y.F., Sun W.J., Sun Y.Y., Li J.L., Zhang J., Wu T., Song T.T., Yao Y.C., Tian J. (2022). MPK6-mediated HY5 phosphorylation regulates light-induced anthocyanin accumulation in apple fruit. Plant Biotechnol. J..

[98] Yang T., Ma H.Y., Li Y., Zhang Y., Zhang J., Wu T., Song T.T., Yao Y.C., Tian J. (2021). Apple MPK4 mediates phosphorylation of MYB1 to enhance light-induced anthocyanin accumulation. Plant J..

[99] Li S.N., Wang W.Y., Gao J.L., Yin K.Q., Wang R., Wang C.C., Petersen M., Mundy J., Qiu J.L. (2016). MYB75 Phosphorylation by MPK4 Is Required for Light-Induced Anthocyanin Accumulation in Arabidopsis. Plant Cell.

[100] Catalá R., Medina J., Salinas J. (2011). Integration of low temperature and light signaling during cold acclimation response in Arabidopsis. Proc. Natl. Acad. Sci. USA.

[101] Uehara N., Sasaki H., Aoki N., Ohsugi R. (2015). Effects of the Temperature Lowered in the Daytime and Night-time on Sugar Accumulation in Sugarcane. Plant Prod. Sci..

[102] Shen J.Z., Zhang D.Y., Zhou L., Zhang X.Z., Liao J.R., Duan Y., Wen B., Ma Y.C., Wang Y.H., Fang W.P. (2019). Transcriptomic and metabolomic profiling of *Camellia sinensis* L. cv. ‘Suchazao’ exposed to temperature stresses reveals modification in protein synthesis and photosynthetic and anthocyanin biosynthetic pathways. Tree Physiol..

[103] Nguyen N.H., Jeong C.Y., Kang G.H., Yoo S.D., Hong S.W., Lee H. (2015). MYBD employed by HY5 increases anthocyanin accumulation via repression of *MYBL2* in *Arabidopsis*. Plant J..

[104] Stracke R., Favory J.J., Gruber H., Bartelniewoehner L., Bartels S., Binkert M., Funk M., Weisshaar B., Ulm R. (2010). The *Arabidopsis* bZIP transcription factor HY5 regulates expression of the *PFG1/MYB12* gene in response to light and ultraviolet-B radiation. Plant Cell Environ..

[105] Zhang Y.Q., Zheng S., Liu Z.J., Wang L.G., Bi Y.R. (2011). Both HY5 and HYH are necessary regulators for low temperature-induced anthocyanin accumulation in *Arabidopsis* seedlings. J. Plant Physiol..

[106] Xie X.B., Li S., Zhang R.F., Zhao J., Chen Y.C., Zhao Q., Yao Y.X., You C.X., Zhang X.S., Hao Y.J. (2012). The bHLH transcription factor MdbHLH3 promotes anthocyanin accumulation and fruit colouration in response to low temperature in apples. Plant Cell Environ..

[107] Mao W.W., Han Y., Chen Y.T., Sun M.Z., Feng Q.Q., Li L., Liu L.P., Zhang K.K., Wei L.Z., Han Z.H. (2022). Low temperature inhibits anthocyanin accumulation in strawberry fruit by activating FvMAPK3-induced phosphorylation of FvMYB10 and degradation of Chalcone Synthase 1. Plant Cell.

[108] Zhou L.J., Geng Z.Q., Wang Y.X., Wang Y.G., Liu S.H., Chen C.W., Song A.P., Jiang J.F., Chen S.M., Chen F.D. (2021). A novel transcription factor CmMYB012 inhibits flavone and anthocyanin biosynthesis in response to high temperatures in chrysanthemum. Hortic. Res..

[109] Liu Y.H., Lin-Wang K., Espley R.V., Wang L., Li Y.M., Liu Z., Zhou P., Zeng L.H., Zhang X.J., Zhang J.L. (2019). StMYB44 negatively regulates anthocyanin biosynthesis at high temperatures in tuber flesh of potato. J. Exp. Bot..

[110] Zipor G., Duarte P., Carqueijeiro I., Shahar L., Ovadia R., Teper-Bamnolker P., Eshel D., Levin Y., Doron-Faigenboim A., Sottomayor M. (2015). *In planta* anthocyanin degradation by a vacuolar class III peroxidase in *Brunfelsia calycina* flowers. New Phytol..

[111] Movahed N., Pastore C., Cellini A., Allegro G., Valentini G., Zenoni S., Cavallini E., D’Inca E., Tornielli G.B., Filippetti I. (2016). The grapevine VviPrx31 peroxidase as a candidate gene involved in anthocyanin degradation in ripening berries under high temperature. J. Plant Res..

[112] Masclaux C., Valadier M.H., Brugiere N., Morot-Gaudry J.F., Hirel B. (2000). Characterization of the sink/source transition in tobacco (*Nicotiana tabacum* L.) shoots in relation to nitrogen management and leaf senescence. Planta.

[113] Avila-Ospina L., Moison M., Yoshimoto K., Masclaux-Daubresse C. (2014). Autophagy, plant senescence, and nutrient recycling. J. Exp. Bot..

[114] Jezek M., Allan A.C., Jones J.J., Geilfus C.M. (2023). Why do plants blush when they are hungry?. New Phytol..

[115] Himelblau E., Amasino R.M. (2001). Nutrients mobilized from leaves of *Arabidopsis thaliana* during leaf senescence. J. Plant Physiol..

[116] Diaz C., Saliba-Colombani V., Loudet O., Belluomo P., Moreau L., Daniel-Vedele F., Morot-Gaudry J.F., Masclaux-Daubresse C. (2006). Leaf Yellowing and Anthocyanin Accumulation are Two Genetically Independent Strategies in Response to Nitrogen Limitation in *Arabidopsis thaliana*. Plant Cell Physiol..

[117] Nemie-Feyissa D., Olafsdottir S.M., Heidari B., Lillo C. (2014). Nitrogen depletion and small R3-MYB transcription factors affecting anthocyanin accumulation in Arabidopsis leaves. Phytochemistry.

[118] Aoyama S., Reyes T.H., Guglielminetti L., Lu Y., Morita Y., Sato T., Yamaguchi J. (2014). Ubiquitin Ligase ATL31 Functions in Leaf Senescence in Response to the Balance Between Atmospheric CO_2_ and Nitrogen Availability in Arabidopsis. Plant Cell Physiol..

[119] Zhang Y.Q., Liu Z.J., Liu J.P., Lin S., Wang J.F., Lin W.X., Xu W.F. (2017). GA-DELLA pathway is involved in regulation of nitrogen deficiency-induced anthocyanin accumulation. Plant Cell Rep..

[120] Larbat R., Olsen K.M., Slimestad R., Lovdal T., Benard C., Verheul M., Bourgaud F., Robin C., Lillo C. (2012). Influence of repeated short-term nitrogen limitations on leaf phenolics metabolism in tomato. Phytochemistry.

[121] Meng J.X., Gao Y., Liu P.Y., Yang C., Shen T., Li H.H. (2020). In vitro Anthocyanin Induction and Metabolite Analysis in *Malus spectabilis* Leaves Under Low Nitrogen Conditions. Hortic. Plant J..

[122] Lv X.M., Zhang Y.X., Hu L., Zhang Y., Zhang B., Xia H.Y., Du W.Y., Fan S.J., Kong L.A. (2020). Low-Nitrogen Stress Stimulates Lateral Root Initiation and Nitrogen Assimilation in Wheat: Roles of Phytohormone Signaling. J. Plant Growth Regul..

[123] Piovan A., Filippini R. (2007). Anthocyanins in *Catharanthus roseus* in vivo and in vitro: A review. Phytochem. Rev..

[124] Zhou L.L., Shi M.Z., Xie D.Y. (2012). Regulation of anthocyanin biosynthesis by nitrogen in TTG1–GL3/TT8–PAP1-programmed red cells of *Arabidopsis thaliana*. Planta.

[125] Simões C., Bizarri C.H.B., Cordeiro L.S., Castro T.C., Coutada L.C.M., Silva A.J.R., Albarello N., Mansur E. (2009). Anthocyanin production in callus cultures of Cleome rosea: Modulation by culture conditions and characterization of pigments by means of HPLC-DAD/ESIMS. Plant Physiol. Biochem..

[126] Lee W.J., Jeong C.Y., Kwon J., Kien V.V., Lee D., Hong S.W., Lee H. (2016). Drastic anthocyanin increase in response to PAP1 overexpression in fls1 knockout mutant confers enhanced osmotic stress tolerance in *Arabidopsis thaliana*. Plant Cell Rep..

[127] Su N.N., Wu Q., Cui J. (2016). Increased Sucrose in the Hypocotyls of Radish Sprouts Contributes to Nitrogen Deficiency-Induced Anthocyanin Accumulation. Front. Plant Sci..

[128] Kaur S., Kumari A., Sharma N., Pandey A.K., Garg M. (2022). Physiological and molecular response of colored wheat seedlings against phosphate deficiency is linked to accumulation of distinct anthocyanins. Plant Physiol. Biochem..

[129] Wang Z.Q., Zhou X.L., Dong L.X., Guo J.N., Chen Y.Y., Zhang Y.Y., Wu L.F., Xu M.J. (2018). iTRAQ-based analysis of the Arabidopsis proteome reveals insights into the potential mechanisms of anthocyanin accumulation regulation in response to phosphate deficiency. J. Proteom..

[130] Pei L.M., Liu J.J., Zhou Y.Y., Jiang Y.H., Li H. (2021). Transcriptomic and metabolomic profiling reveals the protective role of anthocyanins in alleviating low phosphate stress in maize. Physiol. Mol. Biol. Plants.

[131] Tominaga-Wada R., Masakane A., Wada T. (2018). Effect of phosphate deficiency-induced anthocyanin accumulation on the expression of *Solanum lycopersicum GLABRA3* (*SlGL3*) in tomato. Plant Signal. Behav..

[132] Kovinich N., Kayanja G., Chanoca A., Riedl K., Otegui M.S., Grotewold E. (2014). Not all anthocyanins are born equal: Distinct patterns induced by stress in *Arabidopsis*. Planta.

[133] Zheng H.Z., Wei H., Guo S.H., Yang X., Feng M.X., Jin X.Q., Feng Y.L., Zhang Z.W., Xu T.F., Meng J.F. (2020). Nitrogen and phosphorus co-starvation inhibits anthocyanin synthesis in the callus of grape berry skin. Plant Cell Tissue Organ Cult. (PCTOC).

[134] Henry A., Chopra S., Clark D.G., Lynch J.P. (2012). Responses to low phosphorus in high and low foliar anthocyanin coleus (*Solenostemon scutellarioides*) and maize (*Zea mays*). Funct. Plant Biol..

[135] An J.P., Li H.L., Liu Z.Y., Wang D.R., You C.X., Han Y.P. (2023). The E3 ubiquitin ligase SINA1 and the protein kinase BIN2 cooperatively regulate PHR1 in apple anthocyanin biosynthesis. J. Integr. Plant Biol..

[136] He Y.Q., Zhang X.Y., Li Y.Y., Sun Z.T., Li J.M., Chen X.Y., Hong G.J. (2020). SPX4 interacts with both PHR1 and PAP1 to regulate critical steps in phosphorus-status-dependent anthocyanin biosynthesis. New Phytol..

[137] Huang H., Zhao X.Y., Xiao Q., Hu W.J., Wang P., Luo Y.Y., Xia H., Lin L.J., Lv X.L., Liang D. (2023). Identification of Key Genes Induced by Different Potassium Levels Provides Insight into the Formation of Fruit Quality in Grapes. Int. J. Mol. Sci..

[138] Su X.X., Bai C.H., Wang X.H., Liu H.L., Zhu Y.C., Wei L.P., Cui Z.X., Yao L.X. (2022). Potassium Sulfate Spray Promotes Fruit Color Preference *via* Regulation of Pigment Profile in Litchi Pericarp. Front. Plant Sci..

[139] Dai N., Schaffer A., Petreikov M., Shahak Y., Giller Y., Ratner K., Levine A., Granot D. (1999). Overexpression of Arabidopsis Hexokinase in Tomato Plants Inhibits Growth, Reduces Photosynthesis, and Induces Rapid Senescence. Plant Cell.

[140] Asim M., Guo M., Khan R., Sun Y.G., Du S.S., Liu W.T., Li Y., Wang X.L., Wang M.Y., Shi Y. (2022). Investigation of sugar signaling behaviors involved in sucrose-induced senescence initiation and progression in *N. tabacum*. Plant Physiol. Biochem..

[141] Asim M., Zhang Y., Sun Y.G., Guo M., Khan R., Wang X.L., Hussain Q., Shi Y. (2022). Leaf senescence attributes: The novel and emerging role of sugars as signaling molecules and the overlap of sugars and hormones signaling nodes. Crit. Rev. Biotechnol..

[142] Teng S., Keurentjes J., Bentsink L., Koornneef M., Smeekens S. (2005). Sucrose-specific induction of anthocyanin biosynthesis in Arabidopsis requires the MYB75/PAP1 gene. Plant Physiol..

[143] Hedrich R., Sauer N., Neuhaus H.E. (2015). Sugar transport across the plant vacuolar membrane: Nature and regulation of carrier proteins. Curr. Opin. Plant Biol..

[144] Zhang C., Fu J.X., Wang Y.J., Gao S.L., Du D.N., Wu F., Guo J., Dong L. (2015). Glucose supply improves petal coloration and anthocyanin biosynthesis in *Paeonia suffruticosa* ‘Luoyang Hong’ cut flowers. Postharvest Biol. Technol..

[145] Song X.H., Guo H.H., Liu Y., Wan F.F., Zhang J., Chang X.C. (2020). Effects of salicylic acid and sucrose on pigment content in *Pistacia chinensis* leaves. Sci. Hortic..

[146] Gupta A., Singh M., Laxmi A. (2015). Multiple Interactions between Glucose and Brassinosteroid Signal Transduction Pathways in Arabidopsis Are Uncovered by Whole-Genome Transcriptional Profiling. Plant Physiol..

[147] Qu L.J., Hu D.G., Sun C.H., Zhang Q.Y., An J.P., You C.X., Hao Y.J. (2016). Glucose Sensor MdHXK1 Phosphorylates and Stabilizes MdbHLH3 to Promote Anthocyanin Biosynthesis in Apple. PLoS Genet..

